# Validation of an Intraoperative Visual Assessment System Based on Bone Mechanical Properties for Selection of Cementless Total Knee Arthroplasty in an Asian Cohort

**DOI:** 10.3390/jcm15041405

**Published:** 2026-02-11

**Authors:** Dong Hwan Lee, Dai-Soon Kwak, Yong Deok Kim, Se Heon Lee, Nicole Cho, In Jun Koh

**Affiliations:** 1 Department of Orthopaedic Surgery, Yeouido St. Mary’s Hospital, Seoul 07345, Republic of Korea; ldh850606@naver.com; 2 Department of Orthopaedic Surgery, College of Medicine, The Catholic University of Korea, Seoul 06591, Republic of Korea; seraph622@naver.com (Y.D.K.); dltpgjs202@naver.com (S.H.L.); 3 Catholic Institute for Applied Anatomy, Department of Anatomy, College of Medicine, The Catholic University of Korea, Seoul 06591, Republic of Korea; daisoon@catholic.ac.kr; 4 Joint Replacement Center, Eunpyeong St. Mary’s Hospital, Seoul 03312, Republic of Korea; 5 Hackensack Meridian School of Medicine, 123 Metro Blvd, Nutley, NJ 07100, USA; nicole.cho@hmhn.org

**Keywords:** visual grade, arthroplasty, replacement, knee, biomechanical phenomena, compressive strength, prosthesis retention

## Abstract

**Background/Objectives**: Successful cementless total knee arthroplasty (TKA) requires adequate bone quality. However, reliable tools for intraoperative assessment remain limited. This study aimed to introduce a novel visual grading system for evaluating femoral bone during surgery and to assess its correlation with actual bone mechanical properties and suitability for cementless fixation. **Methods**: We prospectively recruited 193 patients receiving posterior-stabilized TKA. Intraoperatively, femoral cutting surfaces were classified into four visual grades (Excellent, Good, Fair, Poor) considering pore appearance and contour integrity. Femoral bone specimens were harvested during box preparation, and bone mechanical properties were measured through indentation testing. Spearman correlation was used to evaluate the relationship between visual grades and bone mechanical properties. Fisher’s exact test was used to evaluate the distribution pattern of cementless suitable and cemented mandatory classifications across visual grading. Receiver operating characteristic (ROC) analysis was used to evaluate diagnostic accuracy for each visual grade cutoff. **Results**: Visual grade strongly correlated with bone mechanical properties (Spearman’s ρ = 0.881, *p* < 0.01). Cementless suitable cases were predominantly distributed in Good/Excellent visual grades, while cemented mandatory cases were mostly found in Fair/Poor grades. However, 8% of Good visual grade specimens exhibited strength warranting cemented fixation, and 18% of Fair visual grade specimens demonstrated adequate mechanical properties for cementless fixation. Using the Good visual grade as a cutoff threshold, ROC analysis showed excellent diagnostic accuracy (AUC = 0.941) with high sensitivity (89%) and specificity (94%). **Conclusions**: The authors’ novel intraoperative visual assessment system demonstrated significant correspondence to measured bone mechanical properties in the distal femur and showed high accuracy in determining suitability for cementless TKA in Asian individuals. Given the ethnic homogeneity of this cohort, further validation in diverse populations is required to generalize these findings.

## 1. Introduction

Cemented fixation has been the primary fixation method for total knee arthroplasty (TKA). However, with the increasing prevalence of obesity and various other factors, coupled with a growing trend of TKA being performed in younger patients, the demographics of TKA recipients are changing [1,2]. As a result, both the frequency and primary causes of revision TKA are evolving. Aseptic loosening of implants is gradually increasing and becoming a predominant cause [3,4]. In response to these changes, implant manufacturing technology is also advancing. With the emergence of 3D printing technologies, the application of porous metal coating techniques, and the utilization of biological materials such as hydroxyapatite, cementless TKA is regaining attention [5,6]. Its use is gradually increasing, and several research studies suggest promising long-term outcomes [7,8].

Despite technological advancements, the use of cementless implants remains limited. This limitation is primarily due to the risk of early failure, which is likely to occur when cementless TKA is performed in patients with inadequate bone strength [9,10]. Conducting a meticulous assessment of adequate bone strength could significantly reduce the possibility of early failure. However, there is no universally accepted assessment method to date. Preoperative examinations such as Dual-Energy X-ray Absorptiometry (DXA) and computed tomography (CT) are available, but no examination is widely recognized as the gold standard [11]. Moreover, intraoperative measures have only been introduced as arbitrary methods, such as the feeling during sawing or the resistance felt when compressing trabecular bone with the surgeon’s manual palpation, and no definitive method has been established [5,11].

We designed this study to investigate the relationship between our novel intraoperative visual bone assessment system and actual bone mechanical properties. While a previous exploratory study by the authors suggested the potential of a preliminary visual grading system when combined with preoperative CT Hounsfield Units (HU) [12], we designed the current study to validate a more refined and objectified version of the system as an independent, standalone tool. Our research addresses three key objectives: First, we aim to determine the association of our intraoperative visual assessment system with measured bone mechanical properties. Second, we seek to evaluate the distribution pattern of cementless suitable and cemented mandatory classifications across visual grading. Third, we intend to determine the diagnostic performance of our novel intraoperative visual assessment system. Through this comprehensive assessment, we demonstrate the utility of our novel visual grading system in determining appropriate distal femoral bone suitability for cementless fixation.

## 2. Materials and Methods

### 2.1. Study Population

A total of 193 patients who received posterior-stabilized TKA with the Triathlon^®^ knee system (Stryker Inc., Mahwah, NJ, USA) were prospectively recruited. The study protocol was approved by our Institutional Review Board (PC22OISI0068) on 6 May 2022, and all participants provided written informed consent. The demographic data of the 193 enrolled patients are summarized in Table 1. The enrollment period was from May 2022 through May 2024. The primary indication for TKA in all recruited patients was end-stage degenerative knee osteoarthritis (Kellgren-Lawrence grade 3 or 4) that was unresponsive to conservative treatment. Patients with inflammatory arthritis, post-traumatic arthritis, or osteonecrosis were excluded to ensure the methodological robustness and homogeneity of the bone mechanical properties evaluated in this study.

### 2.2. Definition and Reliability of Intraoperative Visual Bone Assessment System

**Figure 1:**
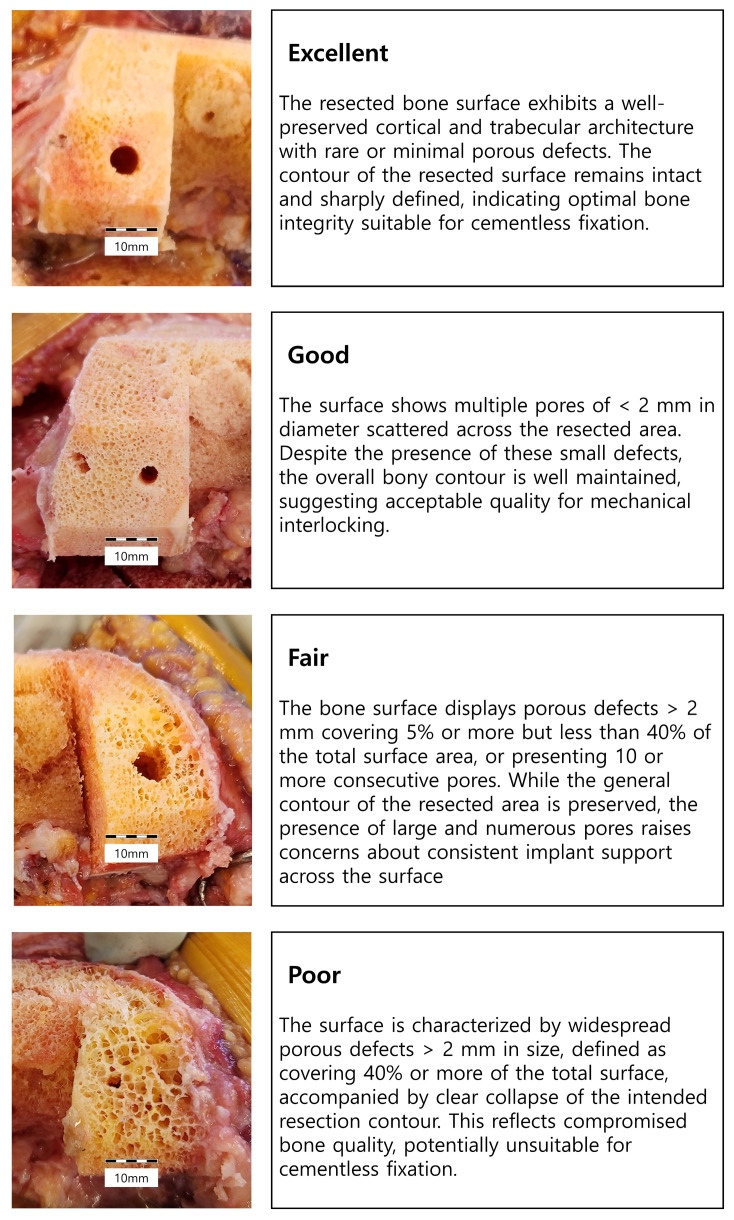
Definition of novel intraoperative visual bone assessment system. Scale bar segments represent 2 mm each (total 10 mm).

The intraoperative visual assessment system was defined based on the morphological features of the prepared femoral bone surface. We categorized cases into four scales: Excellent, Good, Fair, and Poor. Detailed definitions of each grade are described in Figure 1.

We established the 2 mm pore size criterion based on the indentation test’s maximum force application, which produced a 1.8 mm displacement (Figure 1). The percentage-based thresholds for ‘Fair’ and ‘Poor’ grades were established based on biomechanical literature [13]. This study demonstrated that bone volume loss at high-strain locations significantly affects strain distribution and mechanical properties. Specifically, removal of only 4% of bone volume at these critical sites caused considerable changes in strain distribution, while bone volume loss exceeding 7% resulted in a tenfold increase in average strain. Furthermore, a 41% random bone loss throughout the entire bone volume led to a completely flattened strain distribution, severely compromising the bone’s mechanical efficiency and load-bearing capacity. Based on these findings, we set the threshold of 5% to less than 40% for the ‘Fair’ grade and 40% or greater for the ‘Poor’ grade. In actual clinical application, since visual assessment was performed intraoperatively, these percentages were used as approximate reference criteria rather than exact quantified values to guide the grading process. The visual grade was determined independently by the primary surgeon and assistant surgeon at the time of surgery (both experienced arthroplasty surgeons with subspecialty training). All intraoperative assessments were performed after pulsed lavage. Inter-observer reliability was excellent, with an intraclass correlation coefficient (ICC) of 0.91.

### 2.3. Cementless Implant Selection in Clinical Practice

The experimental validation conducted in this study was performed postoperatively using resected box bone specimens obtained during TKA procedures from the enrolled patients. The femoral box bone was selected for mechanical evaluation, as it provides a consistent thickness and volume suitable for standardized indentation testing, which is often not feasible with distal femoral bone fragments. Furthermore, as the box bone represents an area with slightly lower hardness compared to the distal cutting surface located closer to the primary weight-bearing area, its use provides a conservative assessment of bone mechanical properties. This ensures a margin of safety when determining the suitability for cementless fixation based on the intraoperative visual assessment. During surgery, cementless implant selection was determined by applying the criteria of our visual grading system; however, clinical outcome assessment was not the primary objective of this study. This study aimed to validate our intraoperative visual bone assessment system through postoperative biomechanical analysis.

The actual intraoperative implant type selection was performed independently of this experimental study, based on two established criteria. Initially, we utilized HU measurements obtained from routine preoperative CT scans, employing our pre-existing osteoporosis threshold criteria [14]. Second, the operating surgeon directly examined the distal femoral cutting surface during surgery and assessed it according to our visual grading system criteria, with cementless implants used only in cases with a good grade or higher. All enrolled patients underwent a minimum two-year follow-up, with no cases of early implant failure observed in clinical practice.

### 2.4. Bone Mechanical Properties Measurement

**Figure 2:**
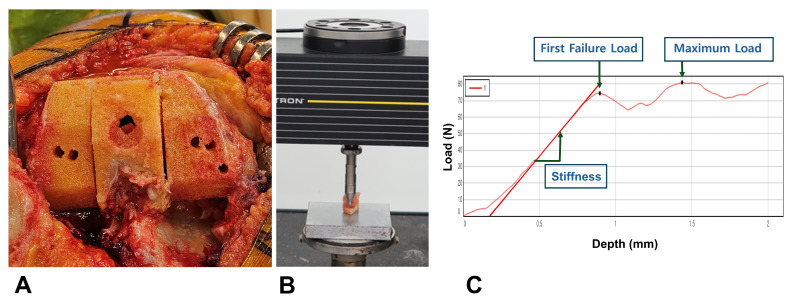
Methodology for bone mechanical properties assessment. (**A**) Box bone specimen collection during posterior-stabilized TKA. (**B**) Indentation testing with a 6 mm thickness specimen. (**C**) Force-displacement curve of the indentation test.

**Figure 3:**
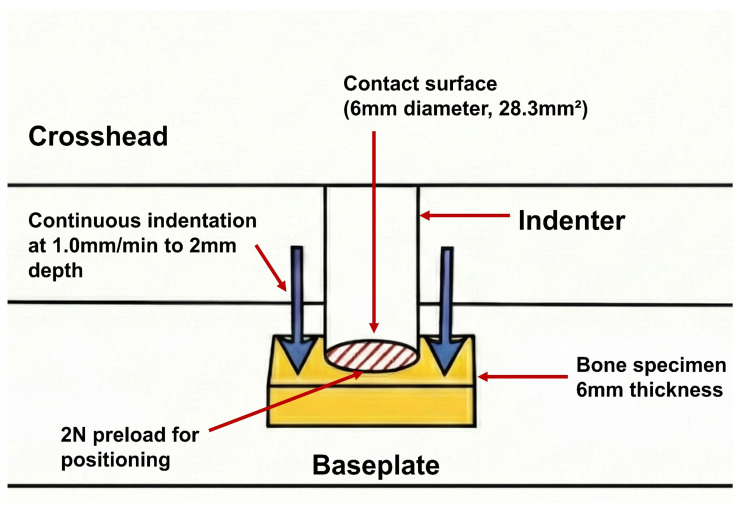
Schematic diagram of the indentation test. The diagram illustrates the interaction between the cylindrical flat-ended indenter (6 mm diameter, 28.3 mm^2^ contact surface) and the 6 mm thick bone specimen.

To quantify bone mechanical properties objectively, we employed a mechanical indentation testing protocol that has been validated in the previous literature across various anatomical sites [15,16,17]. Bone specimens were collected during femoral box preparation and preserved at −70 °C until testing day (Figure 2). After thawing to room temperature, a dual diamond blade saw (IsoMet 5000; Buehler; Lake Bluff, IL, USA) was used to precisely slice specimens to a constant 6 mm thickness. Each specimen underwent testing on a servohydraulic apparatus (Instron 5567; Norwood, MA, USA) using a cylindrical flat-ended indenter (6 mm diameter, 28.3 mm^2^ contact surface). A single indentation test was conducted on each specimen. Due to the constant size of the harvestable box bone specimens, only one test was performed per sample to avoid potential structural interference or micro-damage between multiple indentation sites. Each measurement was performed at a visually uniform area in the central part of the femoral box bone specimen. Furthermore, given the anatomical characteristics of the harvested box bone, all tests were conducted on trabecular bone. The experiment began with a 2 N preload for proper positioning, followed by continuous indentation at 1.0 mm/min to a 2 mm depth (Figure 3). Force-displacement data were captured at 30 Hz and analyzed with Instron Bluehill v4.23 software. The first failure load was our primary outcome measure. This parameter more accurately reflects the point where bone structure begins to fail under load, which is critical for assessing implant stability. The mean first failure load was 59.4 ± 38.1 N (6.7–202.5), occurring at a compressive displacement of 0.9 ± 0.3 mm (0.3–1.8). The maximum load reached 81.0 ± 41.8 N (14.6–244.0) with a corresponding compressive displacement of 1.8 ± 0.3 mm (0.4–2.0). The mean stiffness of the specimen was 110.6 ± 79.9 N/mm (12.0–533.8).

### 2.5. Mechanical Criteria for Cementless TKA Candidacy

**Figure 4:**
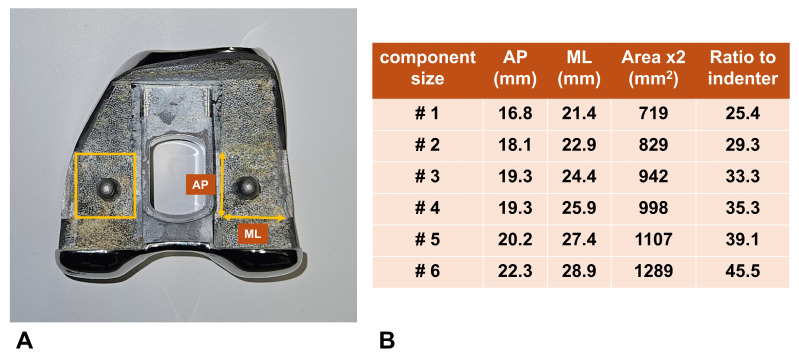
(**A**) Distal surface of TKA femoral implant used for calculation. (**B**) The ratio of the distal surface area to the indenter area (28.3 mm^2^) across implant sizes.

To objectively assess bone suitability for cementless fixation, we developed two key parameters. Based on previous research, we defined the Minimum Required Strength (MRS) as 2.5 times individual body weight, reflecting the typical loading conditions at the knee joint during post-TKA function [18,19]. The Estimated Withstanding Strength (EWS) was also determined for individual specimens by adjusting the first failure load according to the actual implant-bone interface area. This was accomplished by multiplying the first failure load by the ratio between the distal surface of the femoral component and our test indenter area (28.3 mm^2^) (Figure 4). By comparing these values, patients were classified into two categories: those with EWS greater than MRS were deemed “cementless suitable,” while those with EWS less than MRS were considered “cemented mandatory.” Using these criteria, we analyzed the diagnostic accuracy of our novel visual grading system in determining appropriate patients for cementless fixation.

### 2.6. Statistical Analysis

All statistical analyses were conducted with SPSS version 21 (IBM Corp., Armonk, NY, USA). The correlation of visual assessment with first failure load was examined using Spearman’s rank coefficient. The distribution of cementless suitable and cemented mandatory cases across different visual grades was analyzed using Fisher’s exact test, which was chosen due to the presence of cells with expected frequencies of zero. For the diagnostic accuracy assessment, we utilized receiver operating characteristic (ROC) curve analysis in which each level of the visual grading system was treated as a potential cutoff point. For each possible cutoff, we determined the area under the curve (AUC), sensitivity, and specificity to determine which visual grade threshold most accurately predicted bone quality suitable for cementless fixation. A post hoc sample size justification for the primary ROC AUC analysis indicated that the final cohort size (n = 193) was sufficient at α = 0.05 (two-sided) and 80% power. Statistical significance was set at *p* < 0.05.

## 3. Results

**Figure 5:**
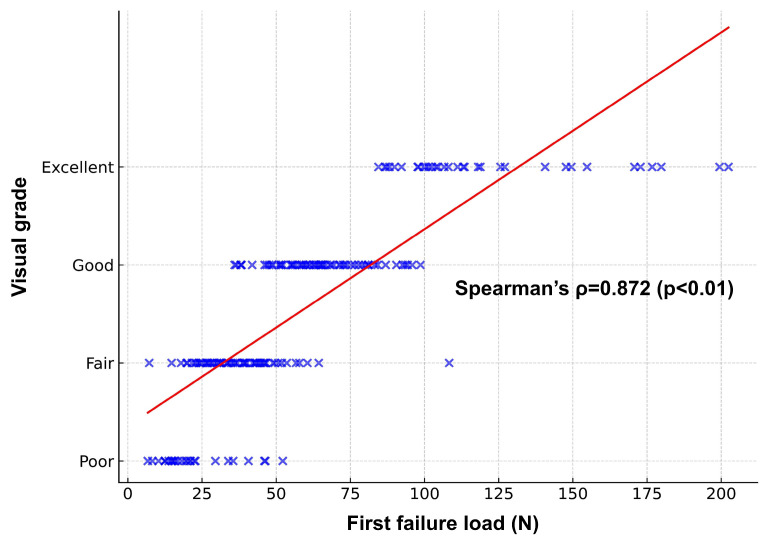
Correlation between visual grades and bone mechanical properties. Scatter plot showing a strong positive relationship (Spearman ρ = 0.881, *p* < 0.01) between visual grades and first failure load measurements.

The visual grade and the first failure load showed a very strong correlation. Spearman’s correlation coefficient (ρ) was 0.881 (*p* < 0.01), indicating a significant positive relationship between the visual grade and the actual distal femoral bone mechanical properties (Figure 5).

Most cases classified as cementless suitable were distributed in the Good and Excellent visual grades, while cemented mandatory cases were predominantly found in the Fair and Poor visual grades (Table 2). Among cases with a Good visual grade, 8% displayed bone mechanical properties that would indicate cemented mandatory, while 18% of cases with a Fair visual grade demonstrated bone mechanical properties sufficient for cementless TKA.

**Figure 6:**
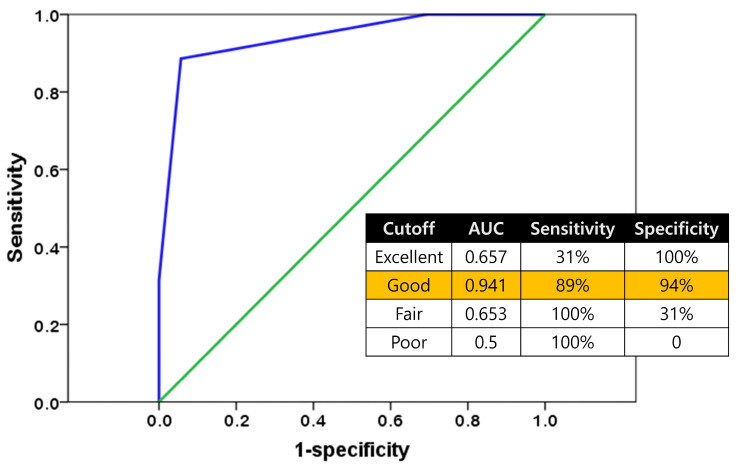
Receiver operating characteristic (ROC) curve analysis for predicting cementless TKA suitability based on visual grading. Selecting the “Good visual grade” as a threshold achieved optimal diagnostic accuracy (AUC = 0.941) with high sensitivity (89%) and specificity (94%), confirming the clinical utility of this intraoperative assessment method for identifying appropriate candidates for cementless fixation.

The visual grade system showed high diagnostic accuracy in identifying suitable distal femoral bone mechanical properties for cementless TKA. Using Good visual grade as the cut-off point, the AUC was 0.941, demonstrating excellent diagnostic value. This cut-off achieved a sensitivity of 89% and a specificity of 94%, reflecting reliable discrimination between adequate and inadequate bone mechanical properties (Figure 6).

## 4. Discussion

Predicting adequate bone strength is essential to prevent early failure in cementless TKA. However, intraoperative assessment methods have not been clearly established [5,11]. Since the bone strength of the cutting surface is the most critical factor for initial fixation of cementless implants, an effective intraoperative assessment method could play a vital role in selecting appropriate candidates for cementless implants. Therefore, we established an intraoperative visual bone assessment system based on the femur cutting surface and investigated its correlation with measured distal femoral bone mechanical properties and its ability to predict suitability for cementless TKA. Unlike our previous research, which utilized a multimodal approach incorporating preoperative imaging [12], this study specifically focuses on validating the independent clinical utility of the refined visual assessment system itself. While the previous exploratory study suggested the potential of a preliminary visual grading system when combined with conventional CT-based HU, the current study was designed to validate a more refined and objectified version as a standalone tool in a larger cohort.

The visual grading demonstrated a strong correlation with the measured distal femoral bone mechanical properties. In our study, the Spearman correlation coefficient (ρ) between visual grade and first failure load was 0.881 (*p* < 0.01), indicating a remarkably strong correlation. Previous intraoperative assessment methods have been vague, such as the feeling during sawing or the resistance felt when compressing trabecular bone with fingers, without clear criteria. We proposed criteria that can function as more objective assessment parameters. This is a preliminary study to compare visual grade with measured bone mechanical properties of the distal femur, conducted only on the femur where bone collection was feasible. Clinically, subsidence has been reported to occur primarily in the tibia [20,21]. Therefore, additional research is needed to determine the actual strength of tibial bone to reflect this clinical reality.

Our data indicate that the majority of cementless suitable cases are classified within the Good or Excellent visual grade, whereas cemented mandatory cases are primarily categorized in the Fair or Poor visual grade. However, 8% of Good visual grade cases exhibited bone mechanical properties adequate for cemented fixation, and 18% of Fair visual grade cases showed bone mechanical properties adequate for cementless fixation. This indicates a potential risk group within the Good visual grade category, comprising 5 out of 193 cases (2.5%) that should be cemented mandatorily. Therefore, additional assessment tools are needed to complement our visual grading approach. We recommend a multi-modal assessment strategy that combines preoperative screening with our intraoperative assessment. Preoperatively, while conventional DXA is widely used, its utility in predicting site-specific bone strength at the knee can be limited [17]. More robust preoperative evaluations should be considered. These include quantitative measurements from DECT or QCT. Recent studies have reported that DECT or QCT can accurately measure bone strength in peripheral joints, including direct measurement of knee joint bone strength, enabling more precise preoperative evaluation [14,22,23]. Even conventional CT-based HU measurements, which are often readily available from routine scans, can serve as a valuable and accessible screening tool. Furthermore, X-ray-based grayscale analysis could also be considered as a low-radiation alternative for assessing bone strength [24]. These preoperative measurement techniques can provide valuable complementary information when combined with our intraoperative visual assessment. Such integration enhances the precision of identifying appropriate candidates for cementless TKA.

Our ROC analysis indicated that selecting the “Good visual grade” as a threshold achieved the highest accuracy, with an AUC of 0.941. This cutoff achieved a sensitivity of 89% and a specificity of 94%, indicating excellent discriminative performance with a low rate of both false negatives and false positives. The thumb test, previously proposed as an intraoperative assessment method, appears to involve more subjective judgment from the surgeon [25]. A previous study reported that its diagnostic accuracy is limited [26]. A method called intraoperative physician assessment (IPA) has been introduced in the knee joint area, which evaluates bone strength based on the resistance felt by the surgeon during the operation [27]. This approach relies on haptic feedback during various preparation processes, such as sawing, drilling, and keeling, but it also requires more subjective judgment from the surgeon. In contrast, our visual grade system has clear visual criteria, reducing subjective judgment and allowing for more objective and consistent grading. Consequently, it demonstrates higher diagnostic accuracy. We believe that its diagnostic value could be further enhanced with the implementation of machine learning or deep learning technologies in the future.

It is essential to clarify the terminology regarding the bone characteristics assessed in this study. The concept of ‘bone quality’ represents a comprehensive and multidimensional construct determined by integrated features across multiple hierarchical levels, including the nano-, micro-, and macro-levels [28,29]. Furthermore, academic discussions have emerged in the bone research field regarding the precision of the term ‘bone quality’ itself, with suggestions that its usage should be reconsidered to avoid ambiguity [30]. In this context, a thorough evaluation of bone quality would require an integrated analysis across all these levels. However, our study focused specifically on bone mechanical properties, particularly the resistance to compressive loading as measured by indentation testing. This focused approach was intentional, as our primary aim was to validate whether a macroscopic intraoperative visual assessment system of the bone cutting surface could reliably predict the risk of early failure in cementless fixation—a clinical challenge primarily governed by local mechanical competence. Therefore, while our visual assessment system provides practical guidance for intraoperative decision-making, it should be clearly understood that this assessment reflects specific mechanical properties rather than the entire spectrum of an individual’s bone quality.

In this study, the visual grading, mechanical testing, and all subsequent analyses were performed exclusively on the distal femur, excluding the tibia. This decision was based on methodological challenges. It was difficult to consistently harvest tibial specimens of sufficient thickness and quality for mechanical testing after bone cutting. Furthermore, tibial cutting specimens often include the dense subchondral bone plate or are in close proximity to the primary weight-bearing surface. These areas posed a high risk of overestimating the true cancellous bone strength of the cutting surface, which could confound the results, and therefore, they were excluded from the experiment. However, although the proximal tibia generally has a lower bone density compared to the distal femur, several previous studies have reported that the bone densities of the two bones are correlated [31,32]. Specifically, significant microarchitectural correlations have been reported. For instance, one study found highly significant correlations between the medial femoral and medial tibial compartments in patients with end-stage osteoarthritis, reporting r-values of 0.97 for porosity and 0.79 for both trabecular number and thickness [31]. Our visual grading system essentially evaluates the trabecular density of the cutting surface, and our study demonstrated that this visual grading correlates strongly with actual distal femoral bone mechanical properties. Given this established microarchitectural correlation between the femur and tibia, it is reasonable to expect that our visual grading system could also be a valuable tool for assessing the tibial cutting surface. Nevertheless, it must be clearly stated that the applicability of our visual grading system to the tibia remains unproven and speculative at this stage. Further biomechanical studies are strictly required to validate the application and accuracy of our grading system on the tibial side.

Despite these positive findings, certain limitations are present in this study. First, our patients were predominantly female (82%) and entirely composed of Asian individuals, reflecting the characteristic population profile among TKA patients from this region [33]. Future research should include more diverse populations across ethnicities and genders to verify whether our findings can be widely generalized to different demographic groups [34]. Second, our analysis pertained exclusively to the Triathlon^®^ knee system (Stryker Inc., Mahwah, NJ, USA). Each manufacturer’s implant has unique structural characteristics that could yield different results when applying our methodology. Therefore, similar analyses should be conducted with various implant systems. Third, our MRS assumption was simplified to 2.5 times body weight, although actual mechanical loading varies among patients and can be influenced by factors such as activity level, alignment, or instability. This simplification poses a risk of misclassification in determining bone suitability. For instance, an underestimation of actual joint loading in highly active patients could lead to a ‘false cementless’ classification, potentially increasing the risk of early implant failure. Conversely, overestimating MRS in sedentary patients might result in ‘false cemented’ decisions, depriving them of the biological fixation benefits of cementless TKA. Consequently, the diagnostic accuracy reported in this study should be interpreted within the constraints of this simplified mechanical model, and future models integrating patient-specific data, such as alignment, activity level, and implant position, are necessary to enhance the precision of identifying suitable candidates for cementless TKA. Finally, while no early implant failure was observed at two-year follow-up, clinical outcomes were not evaluated because the primary aim of this study was to validate the visual grading system through experimental analysis. Having established biomechanical evidence, further clinical evaluation will be necessary in future investigations. While acknowledging these limitations, our findings demonstrate that our intraoperative visual assessment offers an effective means to determine appropriate distal femoral quality for cementless fixation. A major strength of this research lies in its potential clinical application for determining appropriate patients for cementless fixation.

## 5. Conclusions

In this cohort of Asian individuals, the authors’ novel intraoperative visual assessment system demonstrated significant correspondence to measured bone strength in the distal femur and showed high accuracy in determining bone suitability for cementless TKA. Given the ethnic homogeneity of the study population, further validation in diverse ethnic groups is required to generalize these findings. Additionally, while no early failures were observed during the two-year follow-up, the absence of a formal clinical outcome analysis should be noted to avoid overinterpretation of the results.

## Figures and Tables

**Table 1:** Baseline characteristics of the study cohort (n = 193) *.

Characteristic	Values (n = 193)
Demographics	
Age, year	68.2 ± 5.4 [53~86]
Female, n (%)	158 (82)
Height, cm	155.6 ± 7.0 [143.1~177.2]
Weight, kg	65.8 ± 9.6 [45.4~94.3)
Body mass index, kg/m^2^	27.2 ± 3.4 [19.6~36.5)
Bone status by Dual X-ray absorptiometry †	
Normal (T score > −1.0)	Lumbar spine: 115 (61%) Femoral neck: 77 (40%)
Osteopenia (−1.0 ≤ T score ≤ −2.5)	Lumbar spine: 63 (33%) Femoral neck: 93 (49%)
Osteoporosis (T score < −2.5)	Lumbar spine: 12 (6%) Femoral neck: 21 (11%)

* Continuous variables are presented as mean ± SD with range in brackets; categorical variables as n (%); † Dual X-ray absorptiometry thresholds follow the World Health Organization criteria (based on T-scores).

**Table 2:** Distribution based on intraoperative visual bone assessment *.

Visual Grade	Cementless Suitable (n = 105)	Cemented Mandatory (n = 88)	Significance
Excellent (n = 33)	33 (100)	0 (0)	*p* < 0.01
Good (n = 65)	60 (92)	5 (8)	
Fair (n = 68)	12 (18)	56 (82)	
Poor (n = 27)	0 (0)	27 (100)	

* Values represent patient numbers with percentages in parentheses.

## Data Availability

The data that support the findings of this study are available from the corresponding author upon reasonable request.

## Abbreviations

The following abbreviations are used in this manuscript:

TKA	Total knee arthroplasty
ROC	Receiver operating characteristic
AUC	Area under the curve
DXA	Dual-energy X-ray absorptiometry
CT	Computed tomography
ICC	Intraclass correlation coefficient
HU	Hounsfield unit
MRS	Minimum Required Strength
EWS	Estimated Withstanding Strength
DECT	Dual-energy computed tomography
QCT	Quantitative computed tomography
IPA	Intraoperative physician assessment
